# Characterization of a Carbonyl Reductase from *Rhodococcus erythropolis* WZ010 and Its Variant Y54F for Asymmetric Synthesis of (*S*)-*N*-Boc-3-Hydroxypiperidine

**DOI:** 10.3390/molecules23123117

**Published:** 2018-11-28

**Authors:** Xiangxian Ying, Jie Zhang, Can Wang, Meijuan Huang, Yuting Ji, Feng Cheng, Meilan Yu, Zhao Wang, Meirong Ying

**Affiliations:** 1 Key Laboratory of Bioorganic Synthesis of Zhejiang Province, College of Biotechnology and Bioengineering, Zhejiang University of Technology, Hangzhou 310014, China; m15958047548@163.com (J.Z.); m17816035735@163.com (C.W.); meyroline.huang@gmail.com (M.H.); LJ15957189939@163.com (Y.J.); fengcheng@zjut.edu.cn (F.C.); hzwangzhao@163.com (Z.W.); 2 College of Life Sciences, Zhejiang Sci-Tech Univeristy, Hangzhou 310018, China; meilanyu@zstu.edu.cn; 3 Grain and Oil Products Quality Inspection Center of Zhejiang Province, Hangzhou 310012, China

**Keywords:** (*S*)-*N*-Boc-3-hydroxypiperidine, carbonyl reductase, asymmetric reduction, rational design, *Rhodococcus erythropolis*

## Abstract

The recombinant carbonyl reductase from *Rhodococcus erythropolis* WZ010 (ReCR) demonstrated strict (*S*)-stereoselectivity and catalyzed the irreversible reduction of *N*-Boc-3-piperidone (NBPO) to (*S*)-*N*-Boc-3-hydroxypiperidine [(*S*)-NBHP], a key chiral intermediate in the synthesis of ibrutinib. The NAD(H)-specific enzyme was active within broad ranges of pH and temperature and had remarkable activity in the presence of higher concentration of organic solvents. The amino acid residue at position 54 was critical for the activity and the substitution of Tyr54 to Phe significantly enhanced the catalytic efficiency of ReCR. The *k*_cat_/*K*_m_ values of ReCR Y54F for NBPO, (*R*/*S*)-2-octanol, and 2-propanol were 49.17 s^−1^ mM^−1^, 56.56 s^−1^ mM^−1^, and 20.69 s^−1^ mM^−1^, respectively. In addition, the (*S*)-NBHP yield was as high as 95.92% when whole cells of *E. coli* overexpressing ReCR variant Y54F catalyzed the asymmetric reduction of 1.5 M NBPO for 12 h in the aqueous/(*R*/*S*)-2-octanol biphasic system, demonstrating the great potential of ReCR variant Y54F for practical applications.

## 1. Introduction

Many natural products and active pharmaceutical ingredients share a common piperidine core, and the introduction of a chiral hydroxyl group on the C3-position of the piperidine ring may alter the bioactivity of the molecule [1,2,3]. (*S*)-*N*-Boc-3-hydroxypiperidine ((*S*)-NBHP) is a key chiral intermediate in the synthesis of ibrutinib as the inhibitor of Bruton’s tyrosine kinase [4]. In the chemical synthesis of (*S*)-NBHP, employed strategies include the synthesis of racemic 3-hydroxypiperidine followed by chiral resolution and the enantiospecific synthesis of (*S*)-NBHP from chiral precursors. The former only achieves a maximum yield of 50%, making the process economically unviable, while the latter appears to be limited because of the lengthy procedure, rather poor yields of the products, and the use of potentially hazardous reagents [1,5,6]. Alternatively, the carbonyl-reductase-catalyzed asymmetric reduction of *N*-Boc-3-piperidone (NBPO) has gained increasing focus due to its mild reaction conditions, high yield, and remarkable enantioselectivity [4,7,8,9].

Coenzymes are required in carbonyl reductase-catalyzed reactions, and well-established approaches for coenzyme regeneration include the use of a second enzyme and a second substrate (i.e., glucose dehydrogenase and glucose), and the use of the second substrate catalyzed by the same enzyme (i.e., 2-propanol) [10]. Recently, an NADPH-dependent carbonyl reductase from *Saccharomyces cerevisiae* (YDR541C) was employed for the efficient synthesis of (*S*)-NBHP from NBPO by adopting a biphasic system to alleviate product inhibition and using glucose/glucose dehydrogenase to achieve coenzyme regeneration [8]. The glucose/glucose dehydrogenase system yields to the continuous production of gluconic acid; thus, pH adjustment is needed during the reaction, eventually making the process more complex and forming a large quantity of solid waste salt. Alternatively, the 2-propanol oxidation catalyzed by the same carbonyl reductase was widely used for coenzyme regeneration in order to simplify the operating process and increase the solubility of the substrates [11]. An efficient process catalyzed by the commercially-available ketoreductase KR-110 has been demonstrated to reduce 0.5 M NBPO to render the (*S*)-NBHP yield of 97.6% after a 24-h reaction [4]. The enzyme KR-110 was heat-sensitive and the substrate inhibition was obviously observed at a substrate concentration of 0.5 M. In addition, the 2-propanol concentration is usually required in excess to increase the product yield. Thus, high concentrations of the co-substrate together with the substrate further aggravate the inhibition of the enzyme activity in the 2-propanol-coupled strategy [4,11].

To overcome the inhibition from the high load of substrate/co-substrate, protein engineering is one of the promising approaches expanding the upper limit of the substrate/co-substrate concentration on a larger preparative scale [12,13]. Variants of the phenylacetaldehyde reductase from *Rhodococcus* sp. ST-10 (PAR) have been constructed through directed evolution, fully converting 200 g/L ethyl 4-chloro-3-oxobutanoate into ethyl (*S*)-4-chloro-3-hydroxybutyrate in the presence of 15% (*v*/*v*) 2-propanol [14,15]. Furthermore, attempts with biphasic catalysis in the presence of water-immiscible organic solvents have demonstrated an intriguing potential for overcoming the inhibition from substrate/co-substrate, increasing the solubility of substrates, easy product removal, decreasing the spontaneous hydrolysis of substrate/product, and avoiding unfavorable equilibria [16,17,18,19]. In an aqueous/octanol biphasic system, the biosynthesis process of ethyl (*R*)-4-chloro-3-hydroxybutyrate using a stereoselective carbonyl reductase from *Burkholderia gladioli* was established, in which 1.2 M ethyl 4-chloro-3-oxobutanoate was completely converted to afford ethyl (*R*)-4-chloro-3-hydroxybutyrate through the substrate fed-batch strategy [20]. In addition, the integration of protein engineering and medium engineering can further improve the effectiveness of asymmetric reduction at a high substrate load [20,21,22].

**Scheme 1:**
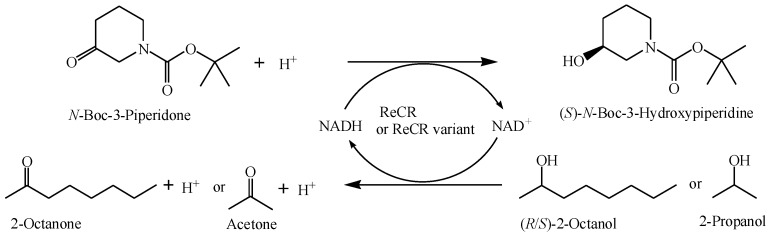
Asymmetric bioreduction of *N*-Boc-3-piperidone (NBPO) using (*R*/*S*)-2-octanol or 2-propanol as co-substrate for NADH regeneration.

Although several processes for the efficient biosynthesis of (*S*)-NBHP have been developed, the pivot carbonyl reductases as biocatalysts still lack an in-depth characterization. Our previous genome mining enabled the discovery of chiral ketoreductases from *Rhodococcus erythropolis* WZ010 and the exploration of its application in the synthesis of chiral alcohols [23,24]. Here, a strictly (*S*)-enantioselective carbonyl reductase from *R. erythropolis* WZ010 (ReCR) and its variant Y54F were characterized for the efficient bioreduction of NBPO to (*S*)-NBHP, providing a basis for process development with an efficient coenzyme regeneration employing (*R*/*S*)-2-octanol or 2-propanol as the co-substrate (Scheme 1).

## 2. Results and Discussion

### 2.1. Characterization of Recombinant ReCR

**Figure 1:**
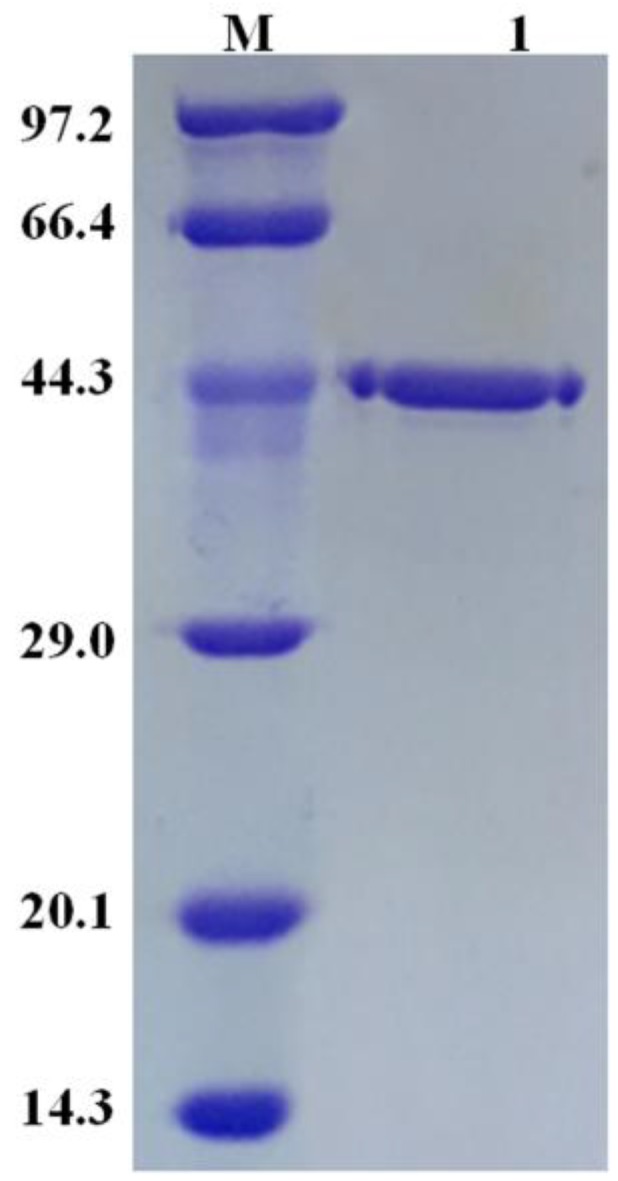
SDS-PAGE (12.5%) analysis of the purified recombinant ReCR. Lane 1, 2 μg purified ReCR with C-terminal His-tag; lane M, molecular weight marker. Coomassie Brilliant Blue R-250 was used to visualize the protein bands in the SDS-PAGE gel.

**Figure 2:**
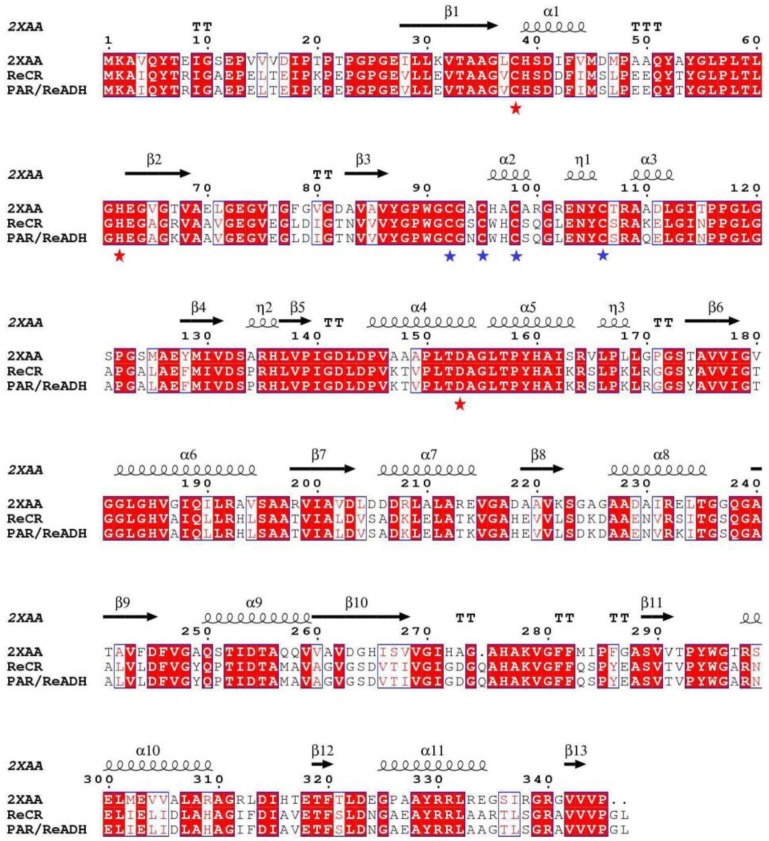
Structure-related sequence alignment between ReCR and its homologous proteins. 2XAA, PDB code of alcohol dehydrogenase from *Rhodococcus ruber* DSM 44541; PAR, alcohol dehydrogenase from *Rhodococcus* sp. ST-10 (GenBank accession No.: AB020760.3); ReADH, alcohol dehydrogenase from *R. erythropolis* DSM 43297 (GenBank accession No.: AY161280.1). The amino acid sequences of both PAR and ReADH are identical. Shown above the alignments are elements of the secondary structure of 2XAA. The numbering shown is from 2XAA. Red stars, putative catalytic residues; blue stars, residues for the coordination of structural zinc. Strictly conserved residues are highlighted with red boxes.

The 1044-bp-long gene encoding ReCR was PCR-amplified from the genomic DNA of *R. erythropolis* WZ010 and over-expressed in *E. coli* BL21(DE3) in the form of the recombinant plasmid pEASY-E2-*recr*. The recombinant ReCR with C-terminal His-tag was subsequently purified by Ni-NTA chromatography. The gene *recr* encoded 348 amino acids with a deduced mass of 36.17 kDa, and the purified recombinant ReCR was verified with a single band of around 44 kDa by SDS-PAGE (Figure 1). The encoded amino acid sequence of ReCR displayed a 98% identity to that of PAR or alcohol dehydrogenase from *R. erythropolis* DSM 43297 (ReADH) [25,26,27,28], with five amino acids Arg67, Ser94, Lys110, Ser233, and Arg336 in ReCR different from Lys67, Asn94, Gln110, Lys233, and Gly336 in PAR or ReADH (Figure 2). The structure-related sequence alignment revealed that the enzyme belonged to the superfamily of zinc-containing alcohol dehydrogenases and had all conserved residues for the binding of catalytic and structural zinc ions [29]. It should be noted that the activity of the enzyme was severely inhibited by the exogenous zinc ion (Table S1), similar to what was observed with other zinc-containing alcohol dehydrogenases [24,30].

**Figure 3:**
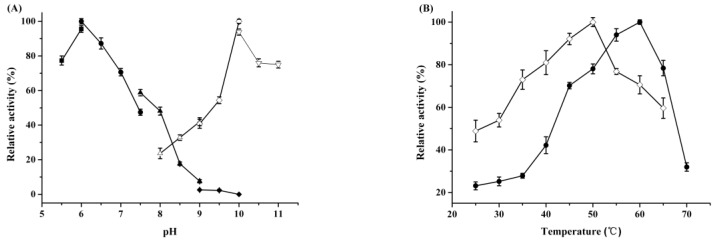
Effect of pH (**A**) and temperature (**B**) on the activity of recombinant ReCR. The relative activities of 100% represent 85.8 U/mg for NBPO reduction (solid symbols) and 88.3 U/mg for (*R*/*S*)-2-octanol oxidation (open symbols). The buffers 2-(*N*-morpholino)ethanesulfonic acid (MES, ■), piperazine-1,4-bisethanesulfonic acid(PIPES, ●), Tris-HCl (▲), and 3-(cyclohexylamino)-2-hydroxy-1-propanesulfonic acid (CAPSO, ◆) were used for the reduction reaction, while the buffers Tris-HCl (△), CAPSO (◇), and 3-(cyclohexylamino)-1-propanesulfonic acid (CAPS, ▽) were used for the oxidation reaction.

**Figure 4:**
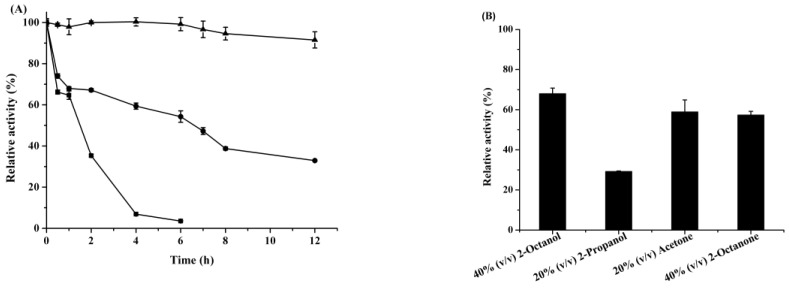
The stability of ReCR against heat (**A**) and organic solvents (**B**). Symbols: (■) for 60 °C, (●) for 55 °C, (▲) for 35 °C. The relative activity of 100% represents 85.8 U/mg for NBPO reduction. The enzyme was incubated with organic solvent (40% (*v*/*v*) (*R*/*S*)-2-octanol, 40% (*v*/*v*) 2-octanone, 20% (*v*/*v*) 2-propanol, or 20% (*v*/*v*) acetone) at 35 °C for 3.5 h prior to the stability test against organic solvent.

The recombinant ReCR was strictly NAD^+^-dependent, since the enzyme activity was not detectable when NADP(H) was used as a coenzyme. The effect of pH on the activity was investigated within the pH range of 5.5–10.5. The maximum activities for NBPO reduction and (*R*/*S*)-2-octanol oxidation were observed at pH 6.0 and 10.0, respectively (Figure 3A), indicating that ReCR-catalyzed oxidation/reduction was pH-dependent [24]. The optimal temperature was 60 °C for NBPO reduction and 50 °C for (*R*/*S*)-2-octanol oxidation (Figure 3B). The enzyme activity in NBPO reduction was stable at 35 °C, whereas the remaining activity decreased to 50% of the initial activity after heat treatment at 60 °C for 1.5 h or 55 °C for 6.5 h (Figure 4A), demonstrating that its thermostability was superior to the heat-sensitive enzyme KR-110 [4]. Among the tested organic solvents, 20% (*v*/*v*) 2-propanol drastically decreased the activity of ReCR, similar to the performance of PAR in the presence of >10% (*v*/*v*) 2-propanol [18]. In contrast to 20% (*v*/*v*) 2-propanol, the enzyme displayed higher stability after 3.5 h incubation with 40% (*v*/*v*) (*R*/*S*)-2-octanol (Figure 4B).

The substrate specificity of ReCR was tested using a set of alcohols and ketones (Table 1). Among the tested substrates, the enzyme exhibited the highest activities with 2,3-butanedione in the ketone reduction and (*R*/*S*)-2-octanol in the alcohol oxidation. The purified ReCR presented an activity of 85.8 U/mg towards NBPO reduction at pH 6.0 and 60 °C. Distinct from PAR and its variants [14], the activity of ReCR toward *N*-Boc-3-pyrrolidone reduction was relatively low. Particularly, the activity towards the oxidation of either (*S*)- or (*R*)-NBHP was not detectable at various temperatures (25–75 °C) and pHs (6.0–10.0), suggesting that the ReCR-catalyzed NBPO reduction was irreversible. A similar case was the secondary alcohol dehydrogenase SdcA from *R. erythropolis* DSM 44534 catalyzing the irreversible (*S*)-2-octanol oxidation [31]. The *K*_m_ and *k*_cat_/*K*_m_ values for NBPO were 1.74 mM and 35.98 s^−1^ mM^−1^, respectively (Table 2). The *k*_cat_/*K*_m_ value for (*R*/*S*)-2-octanol and 2-propanol was 13.04 s^−1^ mM^−1^ and 9.74 s^−1^ mM^−1^, respectively, implying that the use of (*R*/*S*)-2-octanol or 2-propanol as a co-substrate could be feasible to regenerate NADH in the NBPO reduction.

### 2.2. Rational Design and Characterization of ReCR Variant Y54F

For the in-depth characterization, attempts of rational design of ReCR were conducted to improve its activity. The ReCR homology model was built based on the X-ray crystal structure of ADH-A from *Rhodococcus ruber* (PDB: 2XAA). Sequence identity of ReCR towards ADH-A was 60%. The QMEAN and Z-score values were used for the quality evaluation of the models. The QMEAN and Z-score values of the ReCR homology model were 0.822 and 0.533, respectively, which indicated satisfactory quality. In Ramachandran Plot analysis, 91.5% of residues were located in a favorable region, and only 0.4% were found in the sterically disallowed region. This ReCR homology model was selected for subsequent docking studies.

**Figure 5:**
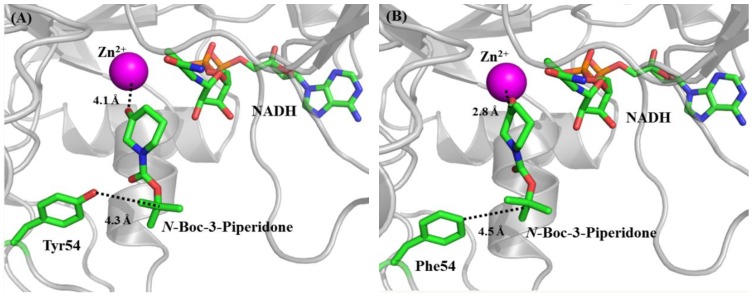
Protein-ligand structures of ReCR with NBPO (**A**) and ReCR Y54F with NBPO (**B**). ReCR and ReCR Y54F are represented in cartoon format. Tyr54, Phe54, NADH, and NBPO are highlighted in sticks. The zinc ion is shown as a magenta sphere.

Furthermore, substrate docking was employed to predict potentially beneficial amino acid positions on ReCR. Figure 5A shows that NBPO was ideally accommodated in the ligand binding pocket of ReCR composed by zinc ion, NADH, and Tyr54 (in the vicinity of the entrance to the active site). Similar to the binding mode of ADH-A with the substrate [29], the carbonyl oxygen atom of NBPO in ReCR was bound to the Zn^2+^ ion with a distance of 4.1 Å, and the carbonyl carbon atom was in close proximity to the C4-atom of NADH. Thus, the hydride was transferred onto the *re*-face of the carbonyl group, consistent with the strict (*S*)-enantioselectivity of ReCR. On the other hand, the bulky Boc group of NBPO was close to the hydroxyl group of Tyr54 (distance of 4.3 Å between the hydroxyl oxygen of Tyr and the tertiary carbon of the Boc group), which might cause a steric hindrance during the substrate binding (Figure 5). Therefore, Tyr54 was selected to be mutated to Phe.

As anticipated, the substitution of Tyr54 to Phe significantly improved the catalytic performance of ReCR, implying that the amino acid residue at position 54 could be critical for the enzyme activity. In the ketone reduction, the *k*_cat_/*K*_m_ values of ReCR Y54F for NBPO (49.17 s^−1^ mM^−1^), acetone (1.47 s^−1^ mM^−1^), and 2-octanone (53.21 s^−1^ mM^−1^) were 1.37, 1.69, and 1.23 times higher than those of ReCR (35.98 s^−1^ mM^−1^, 0.87 s^−1^ mM^−1^, and 43.28 s^−1^ mM^−1^), respectively (Table 2 and Table 3). In the alcohol oxidation, the *k*_cat_/*K*_m_ values of ReCR Y54F for (*R*/*S*)-2-octanol (56.56 s^−1^ mM^−1^) and 2-propanol (20.69 s^−1^ mM^−1^) were 4.34 and 2.12 times higher than those of ReCR (13.04 s^−1^ mM^−1^ and 9.74 s^−1^ mM^−1^), respectively (Table 2 and Table 3). Although the *K*_m_ value of ReCR Y54F for NBPO (1.74 mM) was similar to that of ReCR, the *K*_m_ values of ReCR Y54F for other tested substrates were lowered to a certain extent.

Consistently with kinetic parameters, the productivity of asymmetric bioreduction of NBPO was significantly enhanced when whole cells overexpressing ReCR Y54F instead of ReCR were used as biocatalyst (Table 4). In contrast to the free enzyme, the use of a whole-cell biocatalyst was chosen because of higher enzyme stability and simpler procedure of biocatalyst preparation [4,11,32]. Both 2-propanol and (*R*/*S*)-2-octanol were investigated as co-substrates for the NADH regeneration. In the presence of 10% (*v*/*v*) 2-propanol, the bioreduction of 0.5 M NBPO catalyzed by whole cells overexpressing ReCR Y54F gave a (*S*)-NBHP yield of 98.08% after 12 h, which was 1.34 times higher than that of ReCR (72.15%). The whole-cell biphasic system has been demonstrated to be effective at a higher substrate load, in which (*R*/*S*)-2-octanol instead of 2-propanol was used not only as co-substrate for coenzyme regeneration but also as the organic phase for the substrate reservoir and product sink [33,34]. In the aqueous/(*R*/*S*)-2-octanol biphasic system, the (*S*)-NBHP yield was increased from 77.78% to 95.92% when ReCR Y54F replaced ReCR in the whole-cell biocatalyst. The corresponding total turnover number value of 1199, the calculated space-time yield of 579.15 g L^−1^ day^−1^, and the remarkable stereoselectivity (*e.e.*_p_ > 99.9%) together with the substrate concentration (up to 1.5 M) demonstrated a great potential of ReCR variant Y54F in the practical synthesis of (*S*)-NBHP.

## 3. Materials and Methods

### 3.1. Strain and Growth Condition

The strain *R. erythropolis* WZ010 was deposited in the China Center for Type Culture Collection (CCTCC M 2011336) and used as the donor of the gene *recr* encoding the carbonyl reductase ReCR [35]. The host strains *E. coli* Trans1-T1 and *E. coli* BL21(DE3) were used for the purposes of cloning and over-expression, respectively. Both *R. erythropolis* WZ010 and *E. coli* strains were cultured at 30 °C and 200 rpm for 24 h in Luria-Bertani (LB) medium with a NaCl concentration of 5 g/L, unless stated otherwise.

### 3.2. Construction, Expression, and Purification of Recombinant Enzyme ReCR

The gene *recr* was PCR-amplified from the genomic DNA of *R. erythropolis* WZ010 using forward and reverse primers: *recr*F1 (5′-ATGAAGGCAATCCAGTACAC-3′) and *recr*R1 (5′-CTACAGACCAGGGACCACA-3′). The PCR conditions were listed as follows: denaturalization, 94 °C for 4 min; 30 cycles of 94 °C for 30 s, 53.5 °C for 30 s, and 72 °C for 1 min; and the final extension, 72 °C for 10 min. According to TA cloning strategy from the instructions of the pEASY-E2 expression kit (TransGen Biotech Co., Ltd., Beijing, China), the PCR product was subcloned into the expression vector pEASY-E2 to form the recombinant vector pEASY-E2-*recr* with the C-terminal His-tag. The recombinant plasmid was then transformed into Trans1-T1 competent cells and the recombinant cells were cultured at 37 °C and 200 rpm in LB medium with 100 μg/mL ampicillin (Amp). The recombinant cell named as *E. coli* Trans1-T1/pEASY-E2-*recr* was selected by colony PCRs and the recombinant plasmid pEASY-E2-*recr* was further extracted and verified by DNA sequencing (Sunny Biotechnology, Shanghai, China).

The recombinant plasmid pEASY-E2-*recr* was extracted and then transformed into *E. coli* BL21(DE3) competent cells. The positive recombinant cell named as *E. coli* BL21(DE3)/pEASY-E2-*recr* was cultured at 37 °C and 200 rpm in LB medium with 100 μg/mL Amp. When the OD_600_ reached 0.6, isopropyl β-d-1-thiogalactopyranoside (IPTG) was added to the culture at a final concentration of 0.3 mM, and the temperature was maintained at 20 °C. After 20 h incubation, the *E. coli* cells were harvested by centrifugation and the expression level was analyzed by sodium dodecyl sulfate-polyacrylamide gel electrophoresis (SDS-PAGE). Following the same procedure in the study of 2,3-butanediol dehydrogenase from *R. erythropolis* WZ010 [24], the recombinant ReCR with C-terminal His-tag was purified to homogeneity by nickel affinity chromatography, desalted with 50 mM Tris-HCl (pH 8.0) by ultrafiltration, and stored at −20 °C for further characterization. The subunit molecular mass and purity of ReCR were verified by SDS-PAGE as described previously [36].

### 3.3. Enzyme Activity Assays and Characterization of Recombinant ReCR

The ReCR enzyme activity was measured by the reduction of NAD^+^ or oxidation of NADH at 340 nm (ε_340_ = 6.3 mM^−1^ cm^−1^). Unless otherwise specified, the standard enzyme activity assay for the ketone reduction was performed at 60 °C in duplicate using the assay mixture (2.5 mL) containing 10 mM NBPO, 0.4 mM NADH, and 50 mM PIPES buffer (pH 6.0). The standard assay mixture (2.5 mL) for the alcohol oxidation at 50 °C contained 50 mM (*R*/*S*)-2-octanol, 0.4 mM NAD^+^, and 50 mM CAPSO buffer (pH 10.0). Unless stated otherwise, the reduction and oxidation reactions were initiated by the addition of 5 μg purified enzyme, respectively. One unit of activity was defined as the amount of enzyme that oxidized or reduced 1 μmol NADH or NAD^+^ per minute under optimal pH and temperature. The protein concentrations of ReCR samples were determined using the Bradford reagent with bovine serum albumin as the standard protein.

The optimal temperature of ReCR activity was determined at a series of temperatures ranging from 25 to 70 °C using 50 mM PIPES buffer (pH 6.0) for NBPO reduction or 50 mM CAPSO buffer (pH 10.0) for (*R*/*S*)-2-octanol oxidation. The optimal pH of ReCR activity was determined over a range of pH from 5.5 to 11.0 at 60 °C for NBPO reduction or 50 °C for (*R*/*S*)-2-octanol oxidation. The buffers (50 mM) used were 2-(*N*-morpholino)ethanesulfonic acid (MES, pH 5.5–6.0), piperazine-1,4-bisethanesulfonic acid (PIPES, pH 6.1–7.5), Tris-HCl (pH 7.5–9.0), 3-(cyclohexylamino)-2-hydroxy-1-propanesulfonic acid (CAPSO, pH 9.0–10.0), and 3-(cyclohexylamino)-1-propanesulfonic acid (CAPS, pH 10.0–11.0). All the pH values of the buffers used were determined at 25 °C using a Mettler Toledo FE20 FiveEasy pH Meter (Mettler-Toledo (Schweiz) GmbH, Greifensee, Switzerland).

The thermostability of the ReCR was investigated by determining its residual activities when the enzyme samples were incubated at 35 °C, 55 °C, or 60 °C. To determine the stability in the presence of organic solvents, the enzyme was incubated with organic solvent at 35 °C for 3.5 h and then the residual activities were assayed for NBPO reduction. The determination of kinetic constants for ReCR was carried out using different substrates. The substrates were NBPO (0–20 mM), acetone (0–1 M), 2-propanol (0–70 mM), 2-octanone (0–30 mM), and (*R*/*S*)-2-octanol (0–20 mM). Apparent values of *K*_m_ and *V*_max_ were calculated using a non-linear regression curve fitting to the Michaelis-Menten equation with the software Origin 8.0 (OriginLab Corporation, Northampton, UK). Data of kinetic parameters present mean values ± SD from three independent experiments.

### 3.4. Asymmetric Reduction of NBPO Catalyzed by Whole Cells of E. coli BL21(DE3)/pEASY-E2-recr

The asymmetric reduction of NBPO was carried out using (*R*/*S*)-2-octanol or 2-propanol as a co-substrate for the coenzyme regeneration. In the case of 2-propanol as the co-substrate, the reaction mixture (5 mL) contained 0.5 M NBPO, 10% (*v*/*v*) 2-propanol, 0.4 mM NAD^+^, and 0.4 g wet cells in 50 mM Tris-HCl buffer (pH 8.0). In the aqueous/(*R*/*S*)-2-octanol biphasic system, the reaction mixture (5 mL) contained 1.5 M NBPO, 60% (*v*/*v*) (*R*/*S*)-2-octanol, 1.2 mM NAD^+^, and 1.2 g wet cells in 50 mM Tris-HCl buffer (pH 8.0). The reactions were carried out in a C76 Water Bath Shaker (New Brunswick, Edison, NJ, USA) at 35 °C and 300 rpm for 12 h.

The reaction mixture was extracted with 5 mL of ethyl acetate under strong vibration. The organic phase in the samples was separated by centrifugation and dehydrated with anhydrous sodium sulfate; then, 1 μL dehydrated sample was applied onto the injector (250 °C) for GC analysis. The reactants were determined with an Agilent 6890N (Santa Clara, CA, USA) gas chromatograph equipped with a chiral GC column (BGB174, 30 m × 250 μm × 0.25 μm). The temperature program for GC analysis was set as follows: 5 °C/min from 100 °C to 125 °C, hold 3 min; 2 °C/min to 140 °C, hold 8 min; 1 °C/min to 150 °C. The peak areas were quantitated using specific external standards. The standards NBPO, (*S*)-NBHP, and (*R*)-NBHP were purchased from Sigma-Aldrich Corporation (Shanghai, China). Retention times of the reactants were listed as follows: 26.997 min for NBPO, 28.452 min for (*S*)-NBHP, and 28.739 min for (*R*)-NBHP (Figure S1). Specifically, the (*S*)-NBHP peak was further determined by GC-MS analysis (Figure S2).

### 3.5. Construction, Characterization, and Docking Analysis of ReCR Variant Y54F

Site-specific mutagenesis was carried out by inverse PCR using native pEASY-E2-*recr* as a template and a pair of primers Y54F F1 (5′-TACACCTTCGGCCTTCCTCTCACGC-3′) and Y54F R1 (5′-AAGGCCGAAGGTGTACTGCTCCTCG-3′) under conditions as follows: denaturation, 95 °C for 2 min; 30 cycles of 95 °C for 20 s, 68 °C for 20 s, and 72 °C for 3 min; and the final extension, 72 °C for 8 min. The PCR product was digested at 37 °C for 2 h to digest the native template with the help of *Dpn* I. The digested product was directly transformed into *E. coli* BL21(DE3) competent cells. The positive recombinant cells were cultured at 37 °C and 200 rpm in LB medium with 100 μg/mL Amp. The recombinant cell named as *E. coli* Trans1-T1/pEASY-E2-*recr-mut* was selected by colony PCRs and the recombinant plasmid pEASY-E2-*recr-mut* was further extracted and verified by DNA sequencing (Sunny Biotechnology, Shanghai, China). Following the same procedure for the recombinant ReCR, the positive recombinant cell named as *E. coli* BL21(DE3)/pEASY-E2-*recr-mut* was obtained and the ReCR variant Y54F was purified for further characterization including kinetic parameters and catalytic performance in NBPO reduction.

The homology model of ReCR was built on the X-ray crystallographic structures of ADH-A from *Rhodococcus ruber* (PDB: 2XAA, resolution of 2.8 Å) by HHpred server [37]. Water molecules, ligands, and other hetero atoms (except the NAD^+^ coenzyme and the zinc ion) were removed from the protein molecule. The coenzyme was remodeled as NADH. The charge of the catalytic zinc ion was assigned to +2, and the ligating side chain of Cys 38 was set as deprotonated and negatively charged. For the homology model of ReCR Y54F, the substitution of Tyr54 to Phe was introduced by FoldX [38]. A structure energy minimization of the proteins was performed to remove improper torsions of the side-chain conformation and correct the covalent geometry. The ligand molecule structures (NBPO and NADH) were directly drawn in ChemBioDraw and followed by an energy minimization. Global docking was performed using AutoDock Vina under the default docking parameters [39]. Point charges were initially assigned according to the AMBER03 force field [40], and then damped to mimic the less polar Gasteiger charges. Subsequently, local docking was executed to predict the binding energy and fine-tune the ligand placement in the binding site.

### 3.6. Nucleotide Sequence Accession Number

The nucleotide sequence of ReCR has been submitted to the GenBank database under the accession number of KX827723.

## 4. Conclusions

The enzyme ReCR showed high specific activity, moderate thermostability, and strict (*S*)-stereoselectivity for asymmetric bioreduction of NBPO to (*S*)-NBHP. The NAD(H)-specific enzyme was active over broad pH and temperature ranges, and tolerated a higher concentration of organic solvents, offering greater flexibility in practical biocatalysis. Particularly, the reduction of NBPO to (*S*)-NBHP was irreversible, which was kinetically in favor of both coenzyme regeneration and formation of (*S*)-NBHP. The substitution of Tyr54 to Phe further improved the catalytic efficiency of ReCR including kinetic parameters and the productivity of (*S*)-NBHP. The *k*_cat_/*K*_m_ values of ReCR Y54F for NBPO (49.17 s^−1^ mM^−1^), (*R*/*S*)-2-octanol (56.56 s^−1^ mM^−1^), and 2-propanol (20.69 s^−1^ mM^−1^) were 1.37, 4.34, and 2.12 times higher than those of ReCR (35.98 s^−1^ mM^−1^, 13.04 s^−1^ mM^−1^, and 9.74 s^−1^ mM^−1^), respectively. Furthermore, the (*S*)-NBHP yield was increased from 77.78% to 95.89% in the aqueous/(*R*/*S*)-2-octanol biphasic system when asymmetric reduction of 1.5 M NBPO was catalyzed for 12 h by whole cells of *E. coli* overexpressing ReCR Y54F instead of ReCR. Taken as a whole, ReCR variant Y54F has a great potential in the asymmetric synthesis of (*S*)-NBHP using (*R*/*S*)-2-octanol or 2-propanol as a co-substrate.

## Figures and Tables

**Table 1:** Substrate spectrum of recombinant ReCR against ketones and alcohols ^a^.

Substrate	Relative Activity (%)	Substrate	Relative Activity (%)
*N*-Boc-3-Piperidone	100.0 ^b^ ± 2.6	(*R*/*S*)-2-Octanol	100.0 ^c^ ± 1.6
2,3-Butanedione	189.0 ± 3.4	(*R*/*S*)-2-Pentanol	61.8 ± 2.3
2-Octanone	169.2 ± 2.9	2-Propanol	47.4 ± 0.5
*p*-Bromoacetophenone	143.9 ± 4.3	(*R*/*S*)-2-Butanol	43.8 ± 1.1
Acetoin	47.2 ± 0.7	DL-1-Phenylethanol	31.5 ± 2.1
β-Ionone	34.8 ± 1.2	Cyclohexanol	8.0 ± 1.0
4-Hydroxy-2-butanone	31.8 ± 1.1	2-Buten-1-ol	6.8 ± 0.2
3-Octen-2-one	25.7 ± 0.7	(*S*)-*N*-Boc-3-Pyrrolidinol	2.7 ± 0.4
Acetophenone	25.3 ± 1.0	(*S*)-*N*-Boc-3-Hydroxypiperidine	0
Hydroxyacetone	23.6 ± 0.6	(*R*)-*N*-Boc-3-Hydroxypiperidine	0
*N*-Boc-3-Pyrrolidone	9.2 ± 0.5		
Acetone	4.8 ± 0.3		
2-Bromoacetophenone	1.8 ± 0.1		

^a^ Data present mean values ± SD from two independent experiments. ^b^ Relative activity of 100% represents 85.8 U/mg for NBPO reduction at pH 6.0 and 60 °C; ^c^ Relative activity of 100% represents 88.3 U/mg for (*R*/*S*)-2-octanol oxidation at pH 10.0 and 50 °C.

**Table 2:** Kinetic parameters of recombinant ReCR ^a^.

Substrate	Coenzyme (mM)	*V*_max_ (U mg^−1^)	*K*_m_ (mM)	*k*_cat_ (s^−1^)	*k*_cat_/*K*_m_ (s^−1^ mM^−1^)
NBPO	NADH (0.4)	103.57 ± 2.46	1.74 ± 0.08	62.61 ± 1.49	35.98 ± 0.86
(*S*)-NBHP	NAD^+^ (0.4)	ND ^b^	ND ^b^	ND ^b^	ND ^b^
Acetone	NADH (0.4)	66.30 ± 3.27	46.06 ± 2.62	40.08 ± 1.98	0.87 ± 0.04
2-Propanol	NAD^+^ (0.4)	23.54 ± 0.27	1.46 ± 0.06	14.22 ± 0.16	9.74 ± 0.11
2-Octanone	NADH (0.4)	235.54 ± 5.95	3.29 ± 0.05	142.38 ± 3.11	43.28 ± 0.95
(*R*/*S*)-2-Octanol	NAD^+^ (0.4)	106.57 ± 2.74	4.94 ± 0.45	64.42 ± 1.66	13.04 ± 0.34

^a^ Data present mean values ± SD from three independent experiments. ^b^ ND, not detectable.

**Table 3:** Kinetic parameters of ReCR variant Y54F ^a^.

Substrate	Coenzyme (mM)	*V*_max_ (U mg^−1^)	*K*_m_ (mM)	*k*_cat_ (s^−1^)	*k*_cat_/*K*_m_ (s^−1^ mM^−1^)
NBPO	NADH (0.4)	140.72 ± 6.52	1.73 ± 0.05	85.07 ± 3.94	49.17 ± 2.28
(*S*)-NBHP	NAD^+^ (0.4)	ND ^b^	ND ^b^	ND ^b^	ND ^b^
Acetone	NADH (0.4)	90.46 ± 1.69	37.32 ± 0.56	54.68 ± 1.02	1.47 ± 0.03
2-Propanol	NAD^+^ (0.4)	35.29 ± 0.88	1.03 ± 0.05	21.32 ± 0.53	20.69 ± 0.51
2-Octanone	NADH (0.4)	273.75 ± 7.58	3.11 ± 0.12	165.48 ± 4.58	53.21 ± 1.47
(*R*/*S*)-2-Octanol	NAD^+^ (0.4)	128.19 ± 3.12	1.37 ± 0.08	77.49 ± 1.88	56.56 ± 1.37

^a^ Data present mean values ± SD from three independent experiments. ^b^ ND, not detectable.

**Table 4:** Asymmetric reduction of *N*-Boc-3-piperidone catalyzed by whole cells overexpressing ReCR or ReCR Y54F ^a^.

Enzyme ^b^	Substrate (M)	Co-substrate (*v*/*v*)	Yield (%)	*e.e.*_p_ (%) ^c^
ReCR	NBPO, 0.5	2-Propanol, 10%	72.15 ± 3.51	>99.9 (*S*)
ReCR Y54F	NBPO, 0.5	2-Propanol, 10%	98.08 ± 1.65	>99.9 (*S*)
ReCR	NBPO, 1.5	(*R*/*S*)-2-Octanol, 60%	77.78 ± 2.23	>99.9 (*S*)
ReCR Y54F	NBPO, 1.5	(*R*/*S*)-2-Octanol, 60%	95.89 ± 2.37	>99.9 (*S*)

^a^ Data present mean values ± SD from two independent experiments. ^b^ Whole cells overexpressing ReCR or ReCR variant Y54F. ^c^ The *e.e.*_p_ value (>99.9%) means that no (*R*)-NBHP peak was detected during GC analyses.

## Supplementary Materials

The following are available online at http://www.mdpi.com/1420-3049/23/12/3117/s1, Figure S1: Gas chromatograph analysis for standards *N*-Boc-3-piperidone (A), (*S*)-*N*-Boc-3-hydroxypiperidine and (*R*)-*N*-Boc-3-hydroxypiperidine; Figure S2: Gas chromatograph-mass spectrometry analysis of (*S*)-*N*-Boc-3-hydroxypiperidine in asymmetric reduction of *N*-Boc-3-piperidone; Figure S3: The Michaelis-Menten kinetics of ReCR; Figure S4. The Michaelis-Menten kinetics of ReCR variant Y54F; Table S1: Effect of metal ions, EDTA, dithiothreitol and sodium iodoacetate on the activity of recombinant ReCR.
