# Supplementary material for: Characterization of a Carbonyl Reductase from Rhodococcus erythropolis WZ010 and Its Variant Y54F for Asymmetric Synthesis of (S)-N-Boc-3-Hydroxypiperidine
Source: Molecules. 2018 Nov 28;23(12):3117. doi: 10.3390/molecules23123117 (PMC6321125; doi:10.3390/molecules23123117)
Supplement: Supplementary file 1 [file molecules-23-03117-s001.pdf]

Supporting Information

Characterization of a Carbonyl Reductase from *Rhodococcus erythropolis* WZ010 and Its Variant Y54F for Asymmetric Synthesis of (*S*)-*N*-Boc-3-Hydroxypiperidine

Xiangxian Ying [1,*], Jie Zhang [1], Can Wang [1], Meijuan Huang [1], Yuting Ji [1], Feng Cheng [1], Meilan Yu [2], Zhao Wang [1] and Meirong Ying [3,*]

[1] Key Laboratory of Bioorganic Synthesis of Zhejiang Province, College of Biotechnology and Bioengineering, Zhejiang University of Technology, Hangzhou 310014, China; E-mails: m15958047548@163.com (J.Z.); m17816035735@163.com (C.W.); meyroline.huang@gmail.com (M.H.); LJ15957189939@163.com (Y.J.); fengcheng@zjut.edu.cn (F.C.)

[2] College of Life Sciences, Zhejiang Sci-Tech Univeristy, Hangzhou 310018, China; E-mail: meilanyu@zstu.edu.cn (M.-L.Y.)

[3] Grain and Oil Products Quality Inspection Center of Zhejiang Province, Hangzhou 310012, China

* Correspondence: yingxx@zjut.edu.cn (X.Y.); hz85672100@163.com (M.Y.);Tel.: +86-571-88320781 (X. Y.)

Contents

Supplementary figures 3

Figure S1. Gas chromatograph analysis for standards *N*-Boc-3-piperidone (A), (*S*)-*N*-Boc-3-hydroxypiperidine and (*R*)-*N*-Boc-3-hydroxypiperidine 4

Figure S2. Gas chromatograph-mass spectrometry analysis of (*S*)-*N*-Boc-3-hydroxypiperidine in asymmetric reduction of *N*-Boc-3-piperidone 6

Figure S3. The Michaelis-Menten kinetics of ReCR 10

Figure S4. The Michaelis-Menten kinetics of ReCR variant Y54F 14

Supplementary table 15

Table S1. Effect of metal ions, EDTA, dithiothreitol and sodium iodoacetate on the activity of recombinant ReCR 15

Supplementary figures

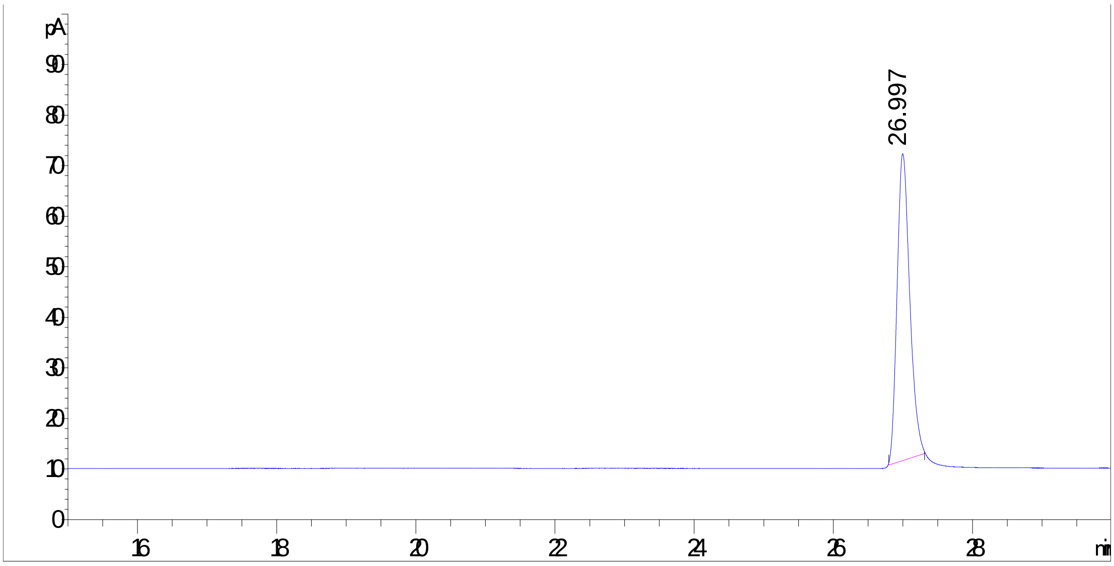

A

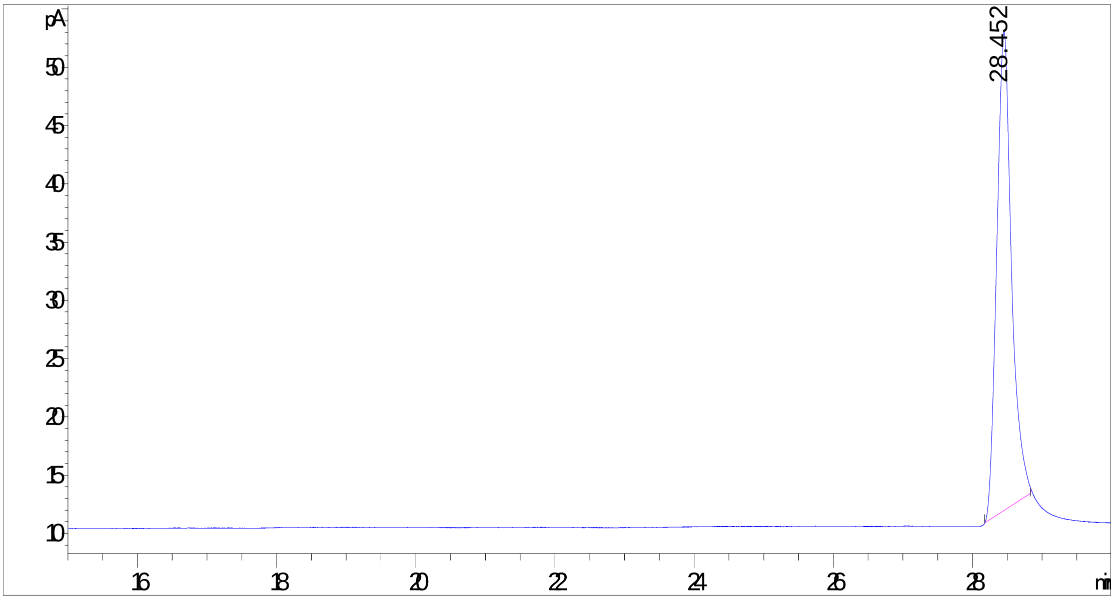

B

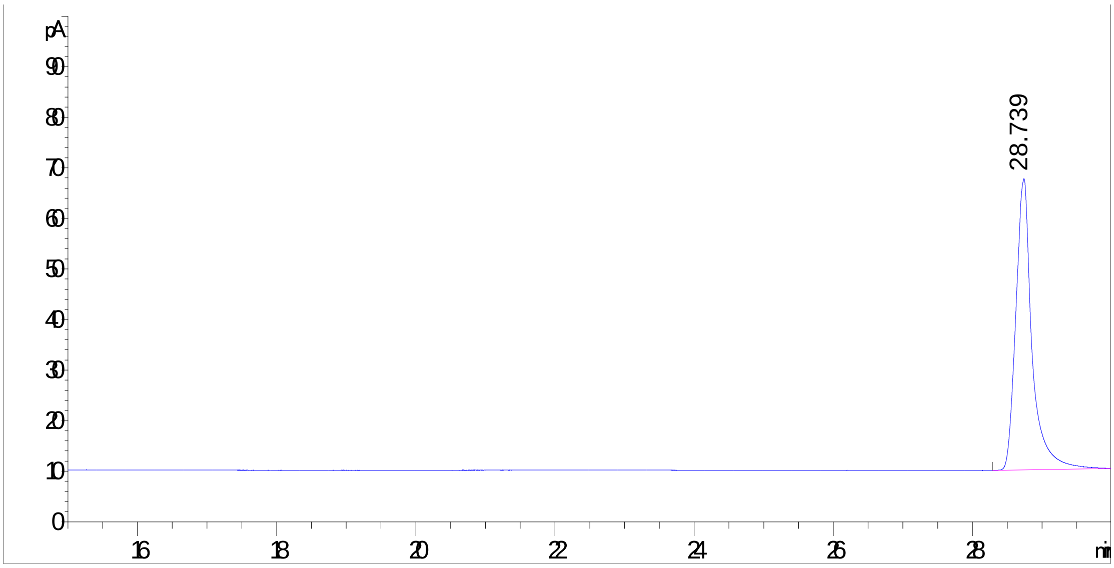

C

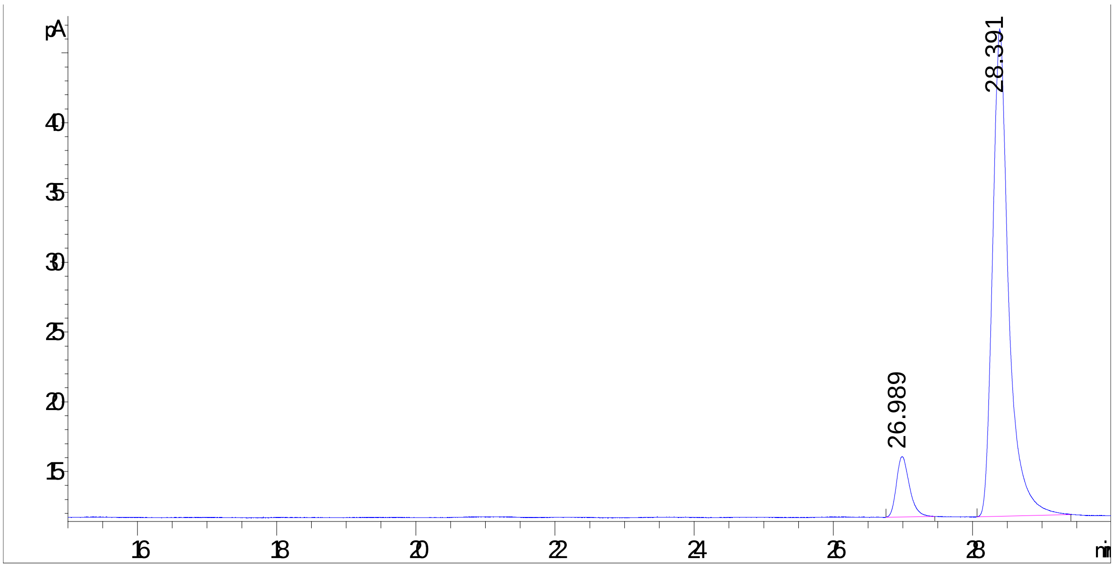

D

Figure S1. Gas chromatograph analysis for standards *N*-Boc-3-piperidone (A), (*S*)-*N*-Boc-3-hydroxypiperidine and (*R*)-*N*-Boc-3-hydroxypiperidine. Figure 1D represents a typical chromatograph of the formation of (S)-*N*-Boc-3-hydroxypiperidine (retention time 28.391 min) from the reduction of *N*-Boc-3-piperidone (26.989).

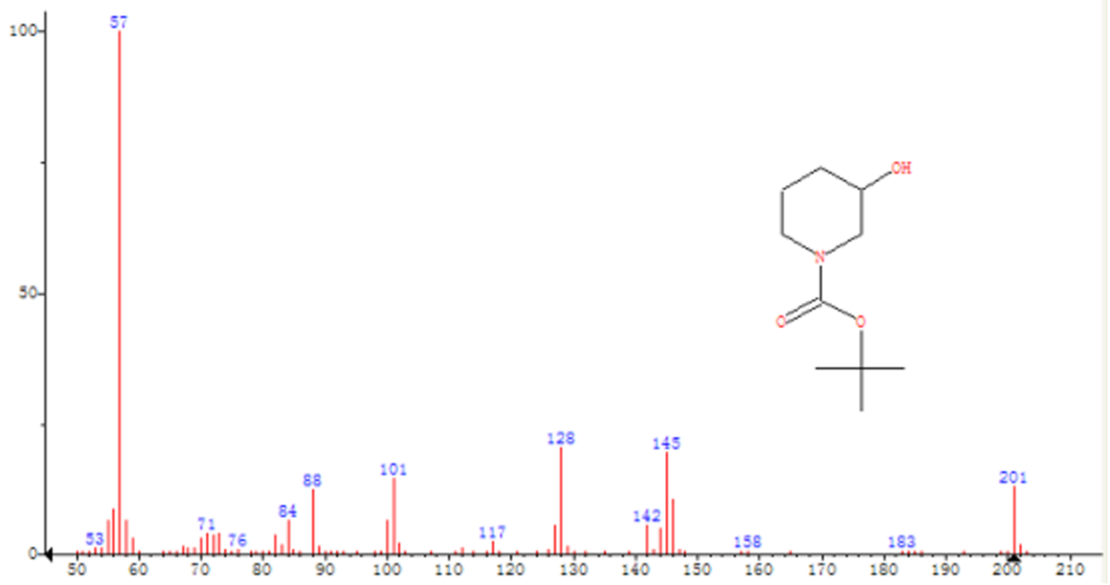

Figure S2. Gas chromatograph-mass spectrometry analysis of (*S*)-*N*-Boc-3-hydroxypiperidine in asymmetric reduction of *N*-Boc-3-piperidone. We analyzed the reaction product by using gas chromatography mass spectrometry (GC–MS; Agilent7890A/5975C, Agilent Technologies, USA). The analysis method of (S)-*N*-Boc-3-hydroxypiperidine comprised the following steps: GC column, BGB174; injection port temperature, 250°C; injection volume, 1 μL; split ratio, 50:1; column flow rate, 1 mL/min; column oven temperature, 5 ℃/min from 100 ℃ to 125 ℃, hold 3 min; 2 ℃/min to 140 ℃, hold 8 min; 1 ℃/min to 150 ℃; auxiliary heating zone temperature, 250 ℃; MS quadrupole temperature,150 ℃; ion source temperature, 230 ℃; scan quality range, 30–500 amu; emission current, 200 μA; and electron energy, 70 eV.

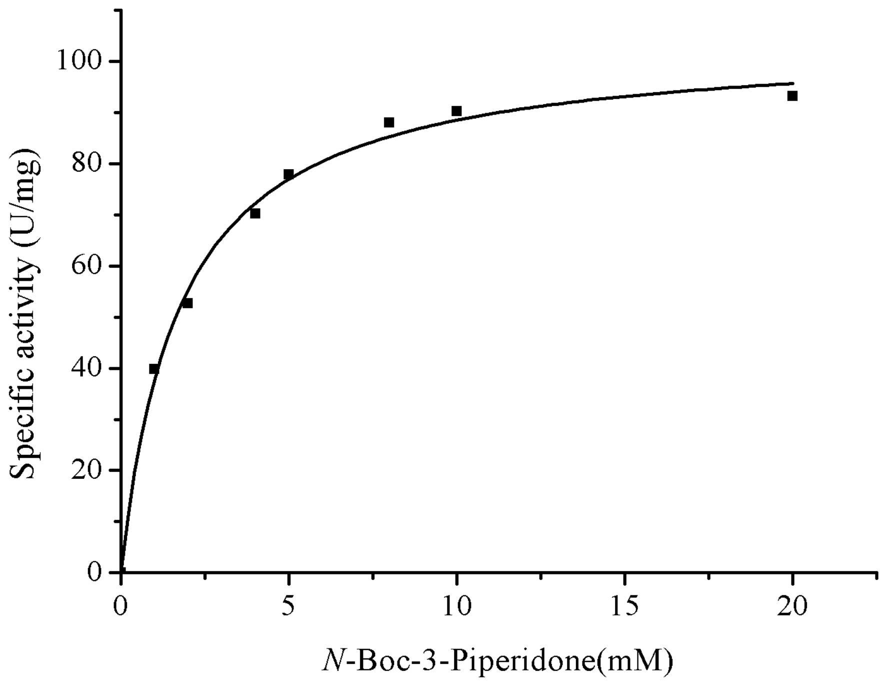
Specific activity (U/mg)
100
80
60
40
20
0
0
5
10
15
20
N-Boc-3-Piperidone(mM)

A

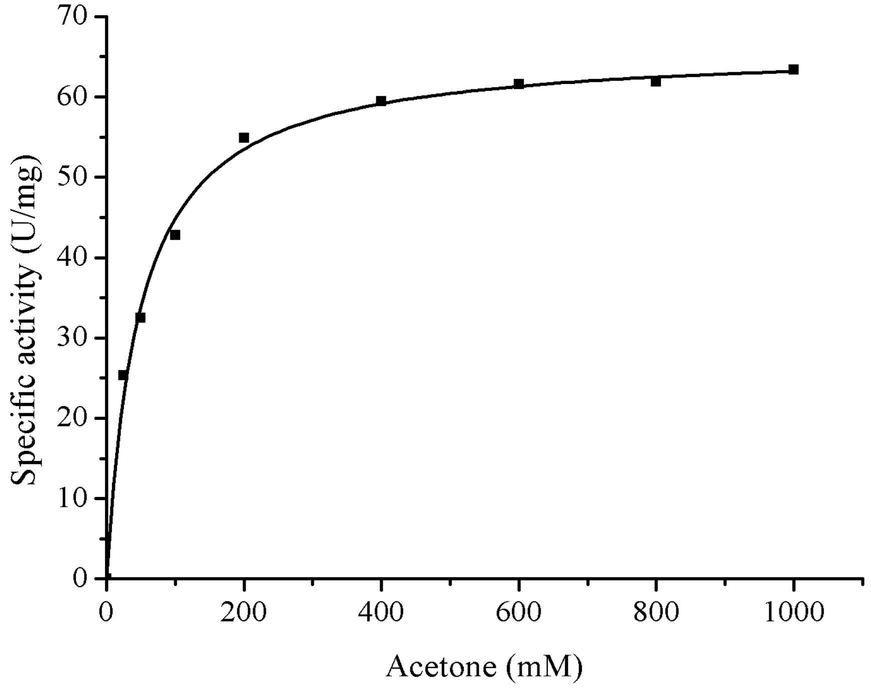
Specific activity (U/mg)
70
60
50
40
30
20
10
0
0
200
400
600
800
1000
Acetone (mM)

B

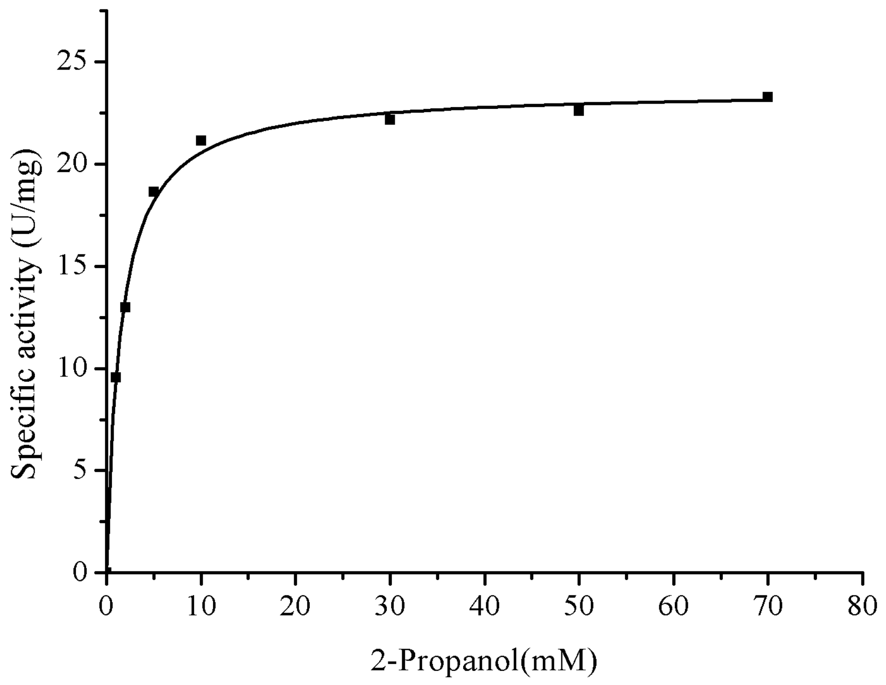

Specific activity (U/mg)
2-Propanol(mM)
0
5
10
15
20
25
0
10
20
30
40
50
60
70
80

C

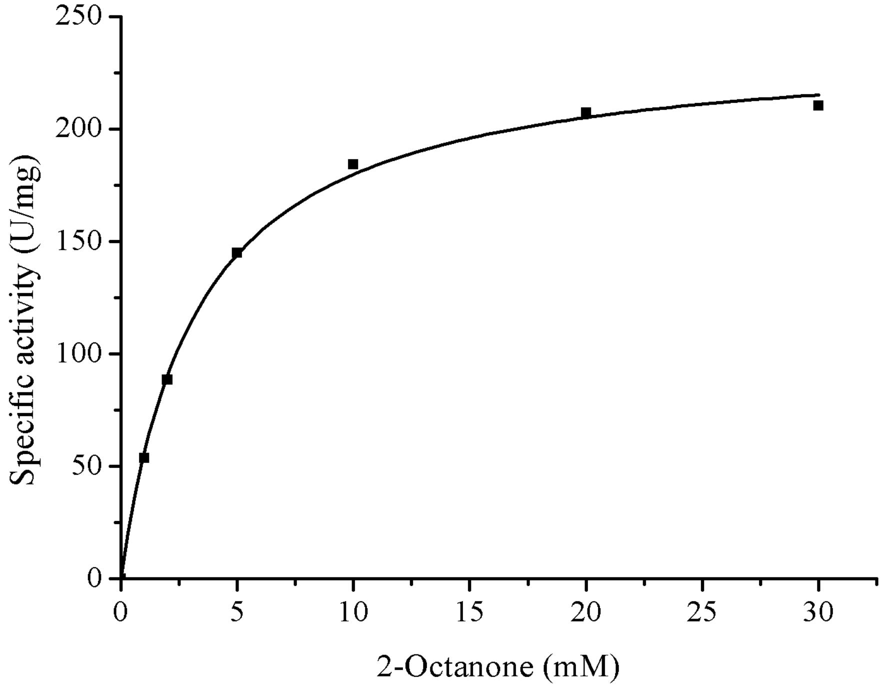
Specific activity (U/mg)
250
200
150
100
50
0
0
5
10
15
20
25
30
2-Octanone (mM)

D

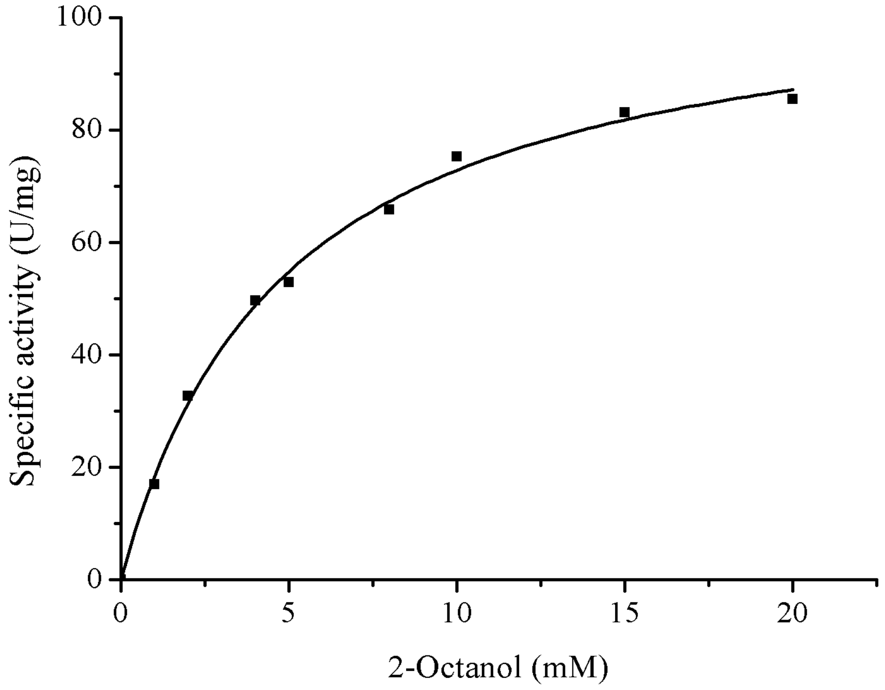

E

Figure S3. The Michaelis-Menten kinetics of ReCR. The tested substrates: A, *N*-Boc-3-piperidone; B, acetone; C, 2-propanol; D, 2-octanone; E, 2-octanol.

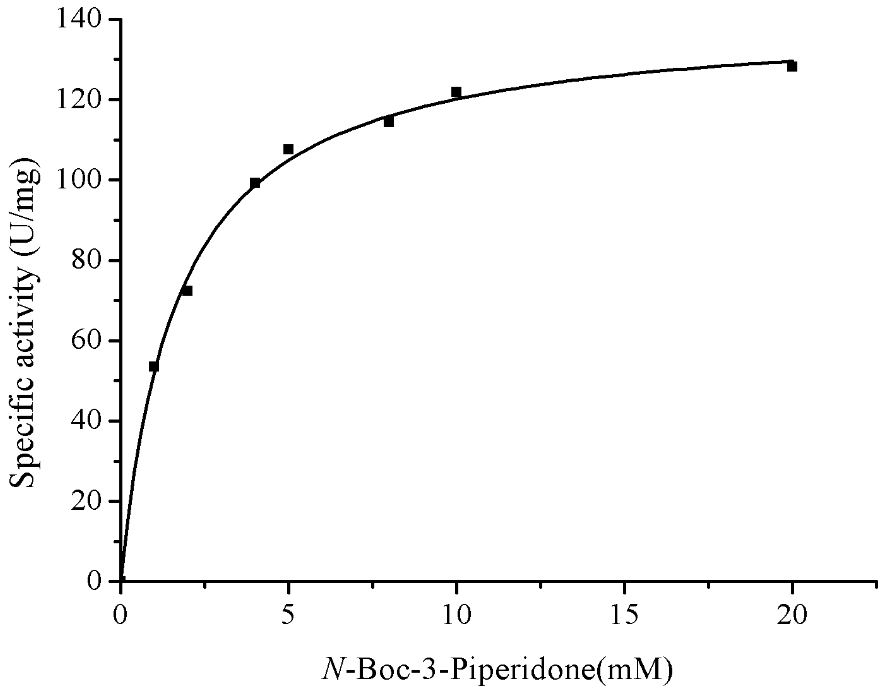

Specific activity (U/mg)
140
120
100
80
60
40
20
0
0
5
10
15
20
N-Boc-3-Piperidone(mM)

A

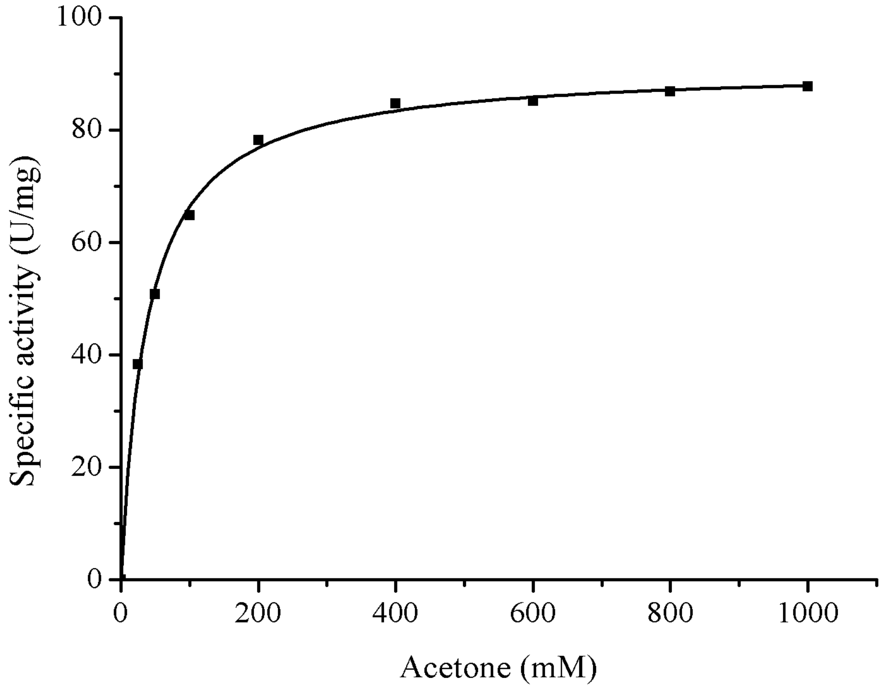
100
80
60
40
20
0
Specific activity (U/mg)
0
200
400
600
800
1000
Acetone (mM)

B

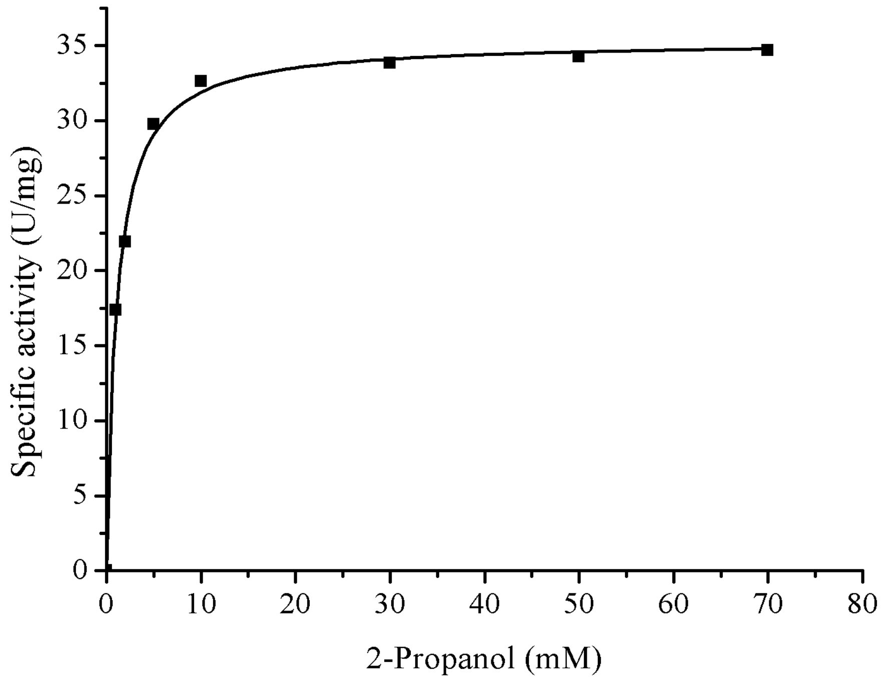
35
30
25
20
15
10
5
0
Specific activity (U/mg)
0
10
20
30
40
50
60
70
80
2-Propanol (mM)

C

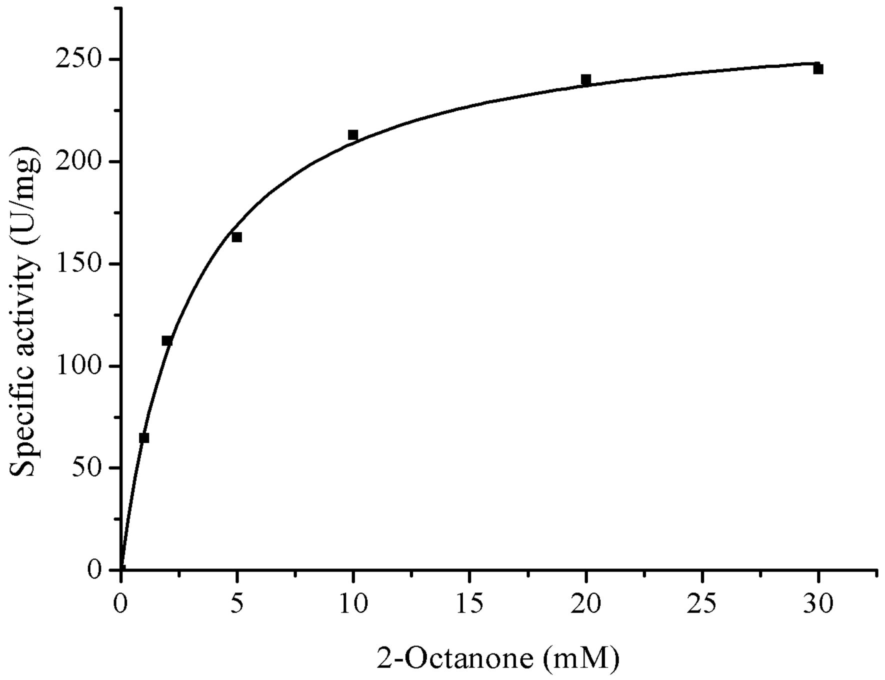

Specific activity (U/mg)
0
50
100
150
200
250
0
5
10
15
20
25
30
2-Octanone (mM)

D

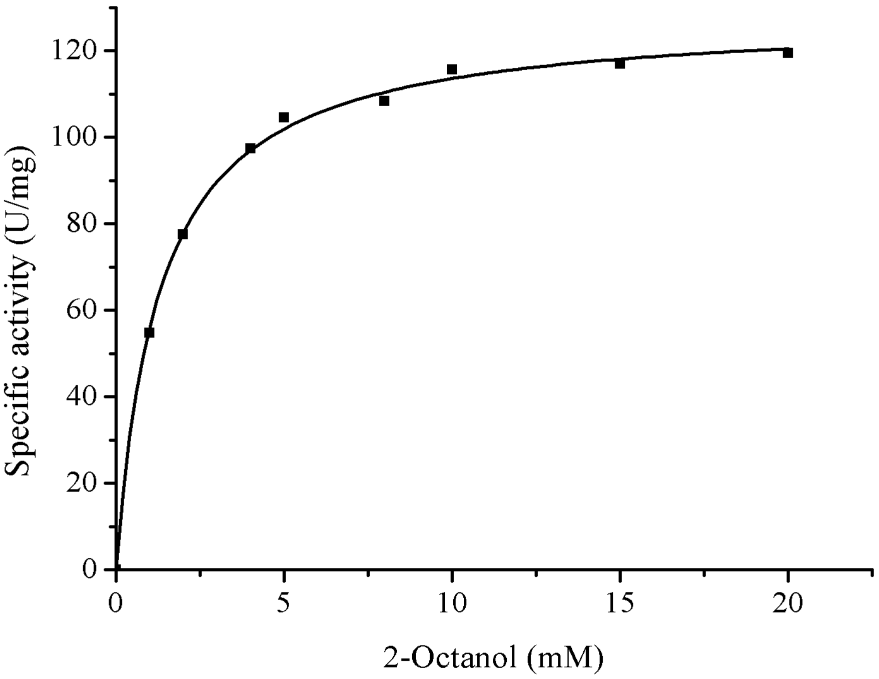

E

Figure S4. The Michaelis-Menten kinetics of ReCR variant Y54F. The tested substrates: A, *N*-Boc-3-piperidone; B, acetone; C, 2-propanol; D, 2-octanone; E, 2-octanol.

Supplementary table

Table S1. Effect of metal ions, EDTA, dithiothreitol and sodium iodoacetate on the activity of recombinant ReCR

Chemicals (mM)	Relative activity (%)
None	100.0[a] ± 2.6
EDTA (1)	144.4 ± 3.7
$CaCl_2$ (1)	116.1 ± 4.2
Dithiothreitol (1)	113.0 ± 1.1
$MnCl_2$ (1)	110.1 ± 0.9
KCl (1)	107.0 ± 1.8
$CoCl_2$ (1)	61.9 ± 2.1
$AlCl_3$ (1)	30.0 ± 0.5
$CuSO_4$ (1)	20.0 ± 0.7
$FeSO_4$ (1)	19.5 ± 0.2
$ZnCl_2$ (1)	13.2 ± 0.5
Sodium iodoacetate (1)	9.7 ± 0.4

[a] The enzyme activity assay was performed at 60℃ in duplicate using the assay mixture (2.5 ml) containing 10 mM *N*-Boc-3-piperidone, 0.4 mM NADH, and 50 mM PIPES buffer (pH 6.0). The relative activity of 100% represents 85.8 U/mg for *N*-Boc-3-piperidone reduction at 60℃ and pH 6.0. Data present mean values ± s.d. from two independent experiments.
